# The Principles and Practice of Medicine

**Published:** 1899-02

**Authors:** 


### Books and Magazines.

THE PRINCIPLES AND PRACTICE OF MEDICINE. By William Osler, M. D., Fellow of the Royal Society; Fellow of the Royal College of Physicians, London; Professor of Medicine in the Johns-Hopkins University, and Physician in Chief to the Johns-Hopkins Hospital, Baltimore, etc., etc. Third edition, entirely revised and enlarged. Published by D. Appleton & Co., New York. Price, cloth, $5.50; leather, $6.00; half morocco, $6.50. Pages, 1181.

It is certainly advisable for us to read attentively the more ephemeral forms of medical literature, but it is not only advisable but indispensable that we should read thoroughly the standard text-books of the day. They are the rich repositories of all the well known and well digested facts of our profession.

In our idler moments it is well to allow our studies to digress into experimental and speculative fields, and from them we may, at long intervals, perhaps return with a sheaf of truth; but in our hours of severe trial, when we are confronted with the graver problems of our responsible calling, it is to the standard text-book we must appeal for guidance. No work on practice can be considered an authority in the same sense as those that are used in the legal profession, but in the sense of containing the usual and accepted facts of current practice there are some authorities, and among those which have written on practice none are preferred before Osler. This, the third edition, has been most thoroughly revised and much enlarged. This enlargement has not much increased the bulkiness or inconvenience of the book, which still remains a portable and well proportioned volume, as the increased contents are conveniently stowed away by a slight increase in the size of page, and decrease in size of type.

The following articles have been entirely rewritten or are new: Vaccination Beri-Beri, The Bubonic Plague, Cerebro-spinal Fever Pneumonia, Malta Fever, Yellow Fever, Dengue, Influenza, Leprosy, The Glandular Fever, The Gonorrheal Infection, Cancer of the stomach, Intestinal neuroses, Jaundice, Diseases of the Bile Passages, Diseases of the Pancreas, Diseases of the Thymus Gland, Diseases of the Spleen, Lymphatism, Addison's Disease, Eucephalitis, Neurasthenia, Erythro-Melalgia, and many shorter articles, as Hypertrophic Stenosis of the Pyloris, Ether Pneumonia, etc. Much new matter has been thrown into almost every chapter, bringing the work fully up to date in every department. I. J. J.

